# Female-biased sex ratios in urban centers create a “fertility trap” in post-war Finland

**DOI:** 10.1093/beheco/arab007

**Published:** 2021-03-18

**Authors:** Jenni E Pettay, Virpi Lummaa, Robert Lynch, John Loehr

**Affiliations:** Department of Social Research, University of Turku, 20014 Turku, Finland; Department of Biology, University of Turku, 20014 Turku, Finland; Department of Anthropology, Pennsylvania State University, State College, PA, USA; Faculty of Biological and Environmental Sciences, Lammi Biological Station, University of Helsinki, 16900 Lammi, Finland

**Keywords:** dispersal, fertility, mate choice, migration, reproduction, sex ratio, urbanization

## Abstract

Because sex ratios are a key factor regulating mating success and subsequent fitness both across and within species, there is widespread interest in how population-wide sex ratio imbalances affect marriage markets and the formation of families in human societies. Although most modern cities have more women than men and suffer from low fertility rates, the effects of female-biased sex ratios have garnered less attention than male-biased ratios. Here, we analyze how sex ratios are linked to marriages, reproductive histories, dispersal, and urbanization by taking advantage of a natural experiment in which an entire population was forcibly displaced during World War II to other local Finnish populations of varying sizes and sex ratios. Using a discrete time-event generalized linear mixed-effects model, and including factors that change across time, such as annual sex ratio, we show how sex ratios, reproduction, and migration are connected in a female-dominated environment. Young childless women migrated toward urban centers where work was available to women, and away from male-biased rural areas. In such areas where there were more females, women were less likely to start reproduction. Despite this constraint, women showed little flexibility in mate choice, with no evidence for an increase in partner age difference in female-biased areas. We propose that together these behaviors and conditions combine to generate an “urban fertility trap” which may have important consequences for our understanding of the fertility dynamics of today including the current fertility decline across the developed world.

## Teaser

We investigate how the local sex ratios (number of men for each woman) affected women’s decisions to marry, reproduce, and disperse after the Second World War in Finland in a population of forced migrants. We find that women in urban areas were less likely to start families when the sex ratio was more female-biased, and women were more likely to move away from rural to urban areas, presumably to pursue opportunities created from urbanization. Together, these behaviors and conditions combine to generate an “urban fertility trap” which may have important consequences for understanding the fertility decline among current urbanizing populations.

## INTRODUCTION

Biased sex ratios in human populations have been implicated in a variety of social problems (Schacht et al. 2014) and have therefore been a focus of research in many disciplines across the biological and social sciences. In human societies, skewed sex ratios can result from excessive male mortality from war (Abramitzky et al. 2011), sex-biased infanticide (Hrdy 1999) or selective abortion (Bongaarts and Guilmoto 2015), and sex-biased migration rates. Population-wide sex ratios have long been recognized as a crucial factor regulating mating success and subsequent fitness both within and across species, and in humans, there has been a focus on how sex ratios affect the likelihood of marriage and family formation. For example, skewed sex ratios can result in the rarer sex having difficulty finding an acceptable partner (Treble and Grossbard-Schechtman 1994). Two opposing hypotheses on the social consequences of an excess of males have been proposed. The first posits that an excess of males generates greater male–male competition which leads to increased violence among males and lower pair-bond stability (Emlen and Oring 1977), while the other hypothesis predicts that a scarcity of females leads to higher male investment in offspring and increased mate guarding (Kokko and Jennions 2008). Although the evidence of the relationship between violence and sex ratio in humans is inconsistent (Schacht et al. 2014), an excess of males at the population level is often associated with more stable relationships and families (Schacht and Kramer 2016) and increased monogamy (Pedersen 1991). These effects are likely to vary between populations due to differences in local ecologies and cultures, however, and therefore need to be studied in different contexts.

While male excess has gained the greatest attention, the impact of female-biased sex ratios on human behavior is generally under-studied (Schacht and Kramer 2016). In theory, female-biased sex ratios will make it more difficult for women to find a monogamous partner, which will either lead to more single women, or later reproduction and ultimately lower parity. For example, after high male mortality during the First World War, French women experienced a “marriage squeeze” whereby they married at older ages which resulted in a decreased age difference between spouses, and women from lower social classes were less likely to find a mate (Abramitzky et al. 2011). From the male perspective, female-biased sex ratios may provide them with an increasing number of opportunities to invest more in mating and less in parental care (Schacht and Borgerhoff Mulder 2015). For example, Abramitzky et al. (2011) found that out-of-wedlock births increased by 1.5 percentage points in regions where male mortality was over 20% as compared to those with 10% male mortality in post-First World War France. One way that women may adjust to a female-biased sex ratio is to lower their partner choice criteria (Lichter et al. 1995). Although individuals are expected to reproduce with the highest quality mate available, highly selective individuals run the risk of either incurring a time cost by waiting until a suitable mate is found or failing to find a mate at all (Gowaty and Hubbell 2009). In human societies with monogamy, the potential costs of vying for the highest quality mate and risking delayed or no reproduction can be especially acute for women because their reproductive window is far more constrained than that of men due to menopause. At the same time, however, women also incur greater energetic and time costs of reproduction (e.g., pregnancy and lactation) than men, and according to predictions derived from parental investment theory this makes their investment in mate quality, and hence offspring quality, particularly important (Andersson 1994). One way to help balance the sex ratios in response to these costs is dispersal. For example, random fluctuations in adult sex ratios due to cohort effects in a South American Savannah Pumé hunter–gatherer groups were mitigated through migration between the groups (Kramer et al. 2017). Because sex-biased dispersal can mediate local mate competition, the observed flexibility in dispersal patterns at sexual maturity in hunter–gatherer populations offers some support for the possibility that these behaviors are adaptive (Moss and Maner 2016). Indeed, Kramer et al. (2017) suggest that sex ratio imbalances led to the formation of alliances between groups in order to facilitate the movement of opposite sex mates. They argued that this laid the foundation for human-typical patterns of network formation and culturally prescribed marriage taboos across local groups.

In general, women living in female-biased populations have the following options: 1) Reduce their mate quality criteria to ensure reproduction by reducing standards for a long-term mate, 2) follow a strategy of opportunistic mating, 3) disperse in hope of finding a mate elsewhere, 4) delay reproduction with the aim of finding a suitable mate later, and 5) foregoing reproduction altogether. These options are of particular relevance for women in many contemporary societies and especially for those residing in urban centers where sex ratios are frequently female-biased. Indeed, the most common environment where women outnumber men is currently found in urban centers around the world. For example, young women are overrepresented in the capital regions of Finland, Hungary and Sweden as well as in most large cities across Germany and Russia (Mrkić et al. 2010; Wiest et al. 2013). Women also outnumber men in urban centers in all Central and South American countries while in these countries rural populations are male-biased (Tacoli 2012).

Previous work has explored many of the potential reasons why urbanization can result in lower fertility (Tacoli 2012; Alves et al. 2013). For instance, it has been posited that rural societies place a higher value on producing children because of their importance for labor, whereas in cities the economic value of children is lower and the costs of raising children higher (e.g., Notestein 1945). It has also been shown that fertility changes can be immediate and studies of migrants from rural to urban centers have shown that migrant fertility rates rapidly adjust to the lower fertility levels of native urban residents (White et al. 2008). Some have attributed this change to cultural transmission as the knowledge, beliefs, and behavior of urban residents affects the recent arrivals and the overall success of family planning programs (Cleland and Wilson 1987; Cleland et al. 1994). Issues surrounding gender equality have also been implicated in fertility declines, and both the freedom of women to make their own choices concerning reproduction as well as the absence of true gender equality that results in women having to choose between career aspirations or family have been cited as being important factors in fertility decline (McDonald 2008). While many of these arguments may yield some insight on a proximate level, approaching the problem from an evolutionary perspective grounded in life-history theory (theory on how natural selection has shaped life cycle of organisms) may better reveal ultimate causes for fertility decline in urban centers. Surprisingly, however, very little research has considered the effect of skewed sex ratios on reproduction despite the fact that it may be an important factor in understanding the effects of urbanization on society.

In urban areas, women not only face increased chances to find work and the concomitant opportunities for education, social advancement, and independence, but continue to bear the primary burden of childbearing and raising children within relationships (Tacoli 2012). The often opposing demands of individual development and reproduction may increase the importance of mate selection and result in a delay in childbearing. More generally, research on fertility in female-biased populations suggests that the onset of reproduction can be either earlier, as found in contemporary western societies (Chipman and Morrison 2013; Uggla and Mace 2016), or later as has been found in a historical population in the United States (Schacht and Smith 2017). These differences are most likely due to societal norms with the delay in onset of reproduction in the historical population likely due to the low acceptability of out of wedlock births. Thus far, research has lacked a broader assessment of the range of options available to women that might influence fertility. For example, fertility researchers have not yet directly examined either flexibility in female mate choice or the possible influence of individual-level dispersal. This is crucial because only research that integrates both of these factors together can convincingly demonstrate how female strategies change and respond to local sex ratios. However, conducting this type of analysis in humans has been difficult either because an experimental approach is not possible, or because the data are compiled and analyzed at an aggregate level which can lead to spurious conclusions (Pollet et al. 2017). Another reason for lack of progress is that women have generally only been assessed within a single community, and the same individuals have not been followed as they experience different environments and sex ratios across their lives.

Here, we use an unusually well-documented data set of the marriages, reproductive histories, movements and occupations of a Karelian population evacuated during World War II from Eastern Finland to other parts of Finland with contrasting local sex ratios and levels of urbanization to test how population sex ratios were associated with parenthood and dispersal behavior in each setting (Loehr et al. 2017; Lynch et al. 2019). Approximately 10% of Finnish territory was ceded to the Soviet Union and approximately 410,000 individuals (12% of the population of Finland at that time) had to flee west (Waris et al. 1952). The resettlement of the Karelian evacuees provides a quasi-natural experiment in which an entire population was differentially distributed to municipalities that varied in their sex ratios, population sizes, and degree of urbanization. This unique historical situation provides an opportunity to investigate how population sex ratios affect the movements and reproduction of young women. A key benefit of this approach is that these evacuees represent a cross section of society, and are unlikely to have had the opportunity to develop strong ties to their destination population, a factor which presumably allowed them greater freedom of movement. In addition to this, the historically low income inequality in post-war Finnish society (Roikonen et al. 2015) combined with the fact that the evacuees had lost much of their possessions, means that the population was relatively homogenous with low stratification of social classes. Finally, we are able to compare rural and urban environments to determine how behavioral decisions might differ between these different environments.

Specifically, we tested whether the local (municipality level) sex ratio influenced women’s decisions to move, their choice of mates and ultimately their reproduction by comparing the age at first birth, age difference between partners and movements of a population of young unmarried women for 10 years after the war (1945–1955). We analyzed women’s decisions in three ways: 1) We tested whether the likelihood of reproducing in a given year is correlated with the sex ratio of a given municipality at that time. A result showing no correlation between sex ratio and the likelihood of reproducing would indicate the possibility that women are accepting any available mate to ensure reproduction. On the other hand, a result indicating a lower probability of reproduction in municipalities with more female-biased sex ratios would suggest that women are foregoing reproduction with less desirable mates (such as those with considerable age difference or already married) and are willing to risk foregoing opportunities to marry with less desirable men for the possibility of finding a higher quality mate in the future. 2) We tested whether absolute age differences of couples varied according to the sex ratio of the municipality in which they lived. Age is one of the most important criteria involved in human mate selection (Conroy-Beam and Buss 2019), and here we assumed that greater variance in the age of couples in areas with female-biased sex ratios would serve as an indication that women are sacrificing mate quality in order to assure reproduction (Ní Bhrolcháin 2001). 3) Finally, we tested whether individuals’ likelihood to disperse was correlated with the sex ratio of the municipalities. A result of a higher probability of migrating away from municipalities with higher female-biased sex ratios would support the idea that women prioritize mate quality and are unwilling to settle for mates available in the municipalities in which they currently live or that they are seeking to avoid highly competitive mating markets in the hopes of finding available or higher quality mates elsewhere. Our data present a unique opportunity to test the relative importance of mate quality, fertility and dispersal by taking advantage of a quasi-natural experiment on a population with low social stratification and relative freedom of movement.

## METHODS

### Data

Our analyses concern Karelian women born in an Eastern Finnish province, Karelia, and relocated to western Finland during the Second World War. We study their life events during 1945–1955. Karelians belonged to the Finnish ethnic group, but spoke their own Karelian dialect of the Finnish language. Religion in Finland was mainly Lutheran, but 14% of the evacuated population professed adherence to the Orthodox faith (Kananen 2013). Finland at the time was largely agrarian and new fields were cleared to resettle the displaced Karelian farmers (Waris et al. 1952). The economic situation was difficult after the war and rebuilding and paying war reparations to Soviet Union affected the economy and society heavily (Pihkala 1999). However, paying reparations and trade between Finland and Soviet Union also increased the speed of industrialization. The years after the war were characterized by high marriage and fertility rates, giving rise to the generation known as “baby boomers” (Karisto 2007). Marriages between the displaced Karelians and locals increased in years following the war suggesting integration (Waris 1952). Fertility rates declined in the 1950s and Finland was urbanizing while women’s labor participation was relatively high compared for example with United States (Sweetser and Piepponen 1967). Lindberg (1955) found that during this time period out of wedlock births were uncommon, but somewhat more prevalent in rural (20 per 1000 births) versus urban (15.3 per 1000 births) environments. Fertility was higher in rural (113.8 per 1000 women aged 20–44 years) versus urban (78.4 per 1000 women aged 20–44 years) environments. Divorce was also uncommon and rates differed significantly between rural and urban environments (e.g., rural = 51 divorces per 100,000 people; urban 216 per 100,000 people in the year 1950).

We take advantage of a massive internal migration event that the Finnish society faced after 30 November 1939, when the Soviet Union invaded Finland and four months later the southern Finnish Karelia—approximately 10% of Finnish territory—was ceded to the Soviet Union forcing approximately 410,000 individuals (12% of the population of Finland at that time) to flee west (Waris et al. 1952). The Finnish government implemented a settlement act to provide land for farmers to replace the territory they had lost in Karelia. Although each village in Karelia was assigned to a specific location in western Finland to keep communities together, people were free to move wherever else they wanted to and frequently did so. Securing farms for evacuees from rural areas took some time, but by 1950 the resettlement process was basically finished (Waris et al. 1952). Post-war sex ratios in Finland were affected by the heavy casualties during the war which varied across communities in part due to differential mortality rates of geographically constructed regiments early in the war (Leskinen and Juutilainen 1999).

A recently digitized MiKARELIA life history database of the Karelian evacuees was obtained from registers called “Siirtokarjalaisten tie” which were compiled from interviews of the Karelian evacuees (Loehr et al. 2017). Interviews took place between 1968 and 1970, and an effort was made to locate everyone who was evacuated from Karelia during the war. Each entry in the registers lists the name, sex, date of birth, birthplace, occupation, year of marriage, reproductive records (name, sex, and date of birth of all children), membership in various organizations, and the years and names of all the places they had lived from birth until the time they were interviewed. If they were married then their name, date of birth, birthplace, and occupation of their spouse were also listed. These books were scanned and software was developed (Kaira Core and Natural Language Processing [NLP] software designed for use with the Finnish language) to digitize and extract these records (see Loehr et al. 2017 for a detailed description of data extraction methods and the MiKARELIA database). Interactive map visualizing movement across the country prior to the war and up until 1967 can be seen in https://tuomassalmi.com/siirtokarjalaiset-visualization/.

Here, we follow the annual reproductive and dispersal decisions of 8296 women from 1945 to 1955 who were born in Karelia, were between the ages of 19 and 42 years during this time period, were unmarried when the war ended in 1945, and whose reproductive status and annual place of residence were known. The entire sample therefore consists of 38,272 person years from 8296 women of whom 6630 had their first child during the study period. To quantify these women’s experiences of locally varying sex ratios across time, we used the number of males/ number of females × 100 for each municipality (*N* = 468) and for each year from 1945 to 1955. These data were compiled from books published by Statistics Finland (Suomen tilastollinen vuosikirja 1946, 1948, 1949, 1950, 1951, 1953, 1954, 1956), in which the total number of all inhabitants and total number of males were recorded each year. Using such annually varying measures of the local sex ratio is important because it provides a robust test of how the same individuals respond to different sex ratios, given that these evacuees were changing their municipality of residence frequently in the years following the war (Waris et al. 1952). Unfortunately, these statistics did not include age categories or marital status at the municipality level, which would be even more ideal data to investigate differences between perceived sex ratio and availability of potential marriage partners. These sex ratios of residence municipalities varied from 60.98 to 126.68. Rural and urban areas differed and towns were more female-biased (rural average sex ratio 92.56 SE ± 7.03; towns’ average sex ratio 79.55 ± 5.15). To adjust for the large variation in the overall size of different municipalities ranging from 66 to 441,345, population size was natural log transformed (Lynch et al. 2019). However, because population size as such does not always correlate with degree of urbanization in Finland (large rural municipalities vs. small towns) and because the sociocultural environment likely differs between urban and rural areas in ways that are not explained by population size (data includes 25 towns and 446 rural municipalities), we also used a dummy variable to indicate whether each municipality was a town (1) or rural (0), based on their status in books by the Statistics Finland (Suomen tilastollinen vuosikirja 1946, 1948, 1949, 1950, 1951, 1953, 1954, 1956).

### Probability of first birth

We first analyzed how the current sex ratio of a woman’s municipality of residence affected her probability to give first birth in a given year using a discrete time-event analysis, implemented in a generalized linear mixed model with binary distribution and logit link function in which reproductive status was the response variable (reproduced (1) vs. not (0) in a given year). In the years spanning the 10 years after the war ended (1945–1955), childless women were entered into the analysis if they were between the ages of 19 and 42 years. After their first birth, they were dropped from the analysis for the following year. First birth rather than marriage was chosen for this analysis, because wedding year was missing for 51% of the sample. However, first birth is a good indicator for family formation, since most children were born within marriage, for example, in 1950 only 5.3% of children were born outside marriage (Suomen tilastollinen vuosikirja 1953). Time varying factors included mean centered age and a quadratic term for mean centered age (as it is expected that the most likely age to have a first birth peaks in the mid to late twenties), sex ratio, and urbanization status (town/rural) based on whether the municipality had a town status, and tested whether the sex ratio affected reproduction differently in these areas by adding an interaction term between municipality type and sex ratio. We also included population size of the municipality of residence and kept population size in the model regardless of statistical significance. Social status was not included because it would drastically limit the sample size to only those women who reported their profession, and a large percentage of the interviewees listed e.g., “wife” or “farmer’s wife” as their occupations and having a profession might be more of a result of not being married rather than something affecting the probability of marriage or reproduction. Statistically insignificant fixed terms that were not our main interest were dropped (*P*-value > 0.05). We also included the following random effects: year to account for general trends in birth rates over time, birth municipality within Karelia to take into account variation due to origin population, and current residency municipality to account for cluster effects of municipalities not explained by the fixed factors we entered into our models.

### Age difference between spouses

Societal norms in the time period studied generally restricted large differences in ages between married couples (Kolk 2015). In general, in Finland at the time, the median age difference between spouses was 2.5 years (Lindberg 1955). However, it is possible that in situations where biased sex ratios reduce the availability of mates norms can shift. Thus, we tested whether women responded to lower sex ratios by marrying a man whose age differed from their own. The sample consisted of 1886 married couples for whom year of marriage was known. Age difference was the absolute value of the difference between a husband’s and wife’s birth year. We ran a generalized linear mixed effects model and entered age difference as a response variable with a negative binomial distribution, because age difference was not normally distributed and contained a high proportion of zeros and small values. For the main predictor, sex ratio, we used the value from the year and the municipality in which she was married. We then added age, quadratic term of age, population size, and type of municipality (town/rural) as fixed terms. We also tested interactions between sex ratio and age, because in areas with more females, older women might have a higher probability of marrying a much younger or older man as a spouse as compared to younger women. We also fitted an interaction between sex ratio and municipality type as rural and more urban areas might have had differing norms and ways to find spouses. Because variance in partner age, rather than absolute age difference in years might be related to population sex ratios, we also extracted residuals from the model and conducted an additional regression analysis between these residuals and sex ratio.

### Dispersal probability

We then analyzed if a lower, and thus more female-biased sex ratio was associated with a higher dispersal probability from the municipality of residence using a discrete time-event analysis, implemented in a generalized linear mixed model with a binary distribution and logit link function. The sample included 30,847 observation years from 7179 women with 4963 events (event = individual moving out from residency municipality). The response variable was coded as 1 if a woman moved out from her municipality during the observation year and 0 if she stayed in the same municipality. Time varying variables were sex ratio, age, population size, and municipality type (rural/town). The sample included 19–42 years old women who had not given birth yet, and were therefore assumed to still look for a spouse. Time varying variables included sex ratio, age, population size, and municipality type (rural/town). In addition, because settling down in new locations is known to have happened gradually after the end of the war, we also included number of years since 1945 as a covariate. Previous number of movements after the war might also affect dispersal decisions and we therefore included a dummy variable to indicate how many peace-time movements a woman already had. This number was skewed; three or more movements were grouped into one category (0 movements: 82,568, 1 movement: 12,041, 2 movements: 3975, and 3 or more movements: 1315 observations). Furthermore, we included percentage of evacuees within the current municipality of residence for the year 1946 (Suomen tilastollinen vuosikirja 1948), because contact with fellow evacuees might affect the probability to disperse. We further included an interaction between sex ratio and municipality type, since sex ratio might have differing effects on movements in urban and rural environments. Because an event (moving out) could happen several times to a single person, person identity was also fitted as arandom effect.

## RESULTS

### Population sex ratio and probability of giving first birth

**Figure 1:**
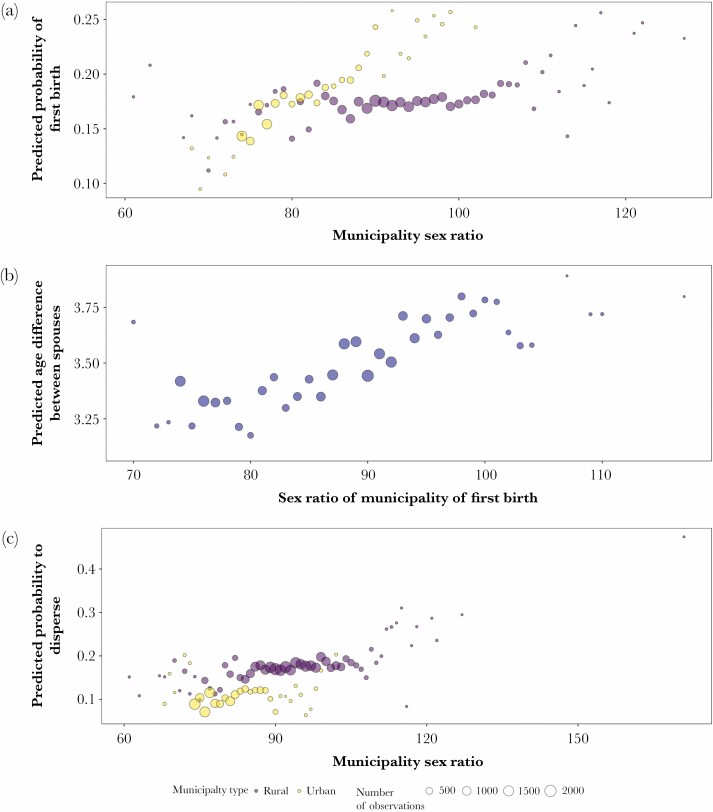
Relationship between dependent variable and sex ratio for each municipality during the years 1945–1955 in Finland. (A) The probability of having a first child in relation to sex ratio in urban and rural communities. (B) Figure illustrating the relationship between the age difference between spouses and the sex ratio of municipality of residence in the year of marriage. (C) The relationship between residence municipality sex ratio and women’s probability to move out in both rural and urban areas. Color code: purple = rural and yellow = urban areas. Probability values are the values predicted by the models. Number of observations are indicated by size of circles.

The mean age at first birth in our sample was 28.5 ± 0.02 years. We found a significant negative interaction between sex ratio and municipality type which suggests that sex ratio was associated with a woman’s probability of giving birth to her first child differently in urban versus rural areas (rural: β = −0.023 ± 0.007, *P* = 0.002, *N* = 38,265 observation years, 6553 first births) (Table 1; Figure 1A.). The model predicts that in rural areas a one unit increase in the sex ratio toward male bias (e.g., from 88 to 89) was related to increases of the odds of having one’s first child by 0.4%, while in towns these odds increased by 2.7%. This indicates that in urban environments, women were especially more likely to reproduce for the first time when there were more men. The effect of squared age was also a significant predictor which suggests that there is a nonlinear trend in the age at first birth for women between the ages of 19 and 42 years (β = −0.005 ± 0.0005, *P* < 0.0001). Population size was not related to the probability of giving first birth (β = −0.005 ± 0.02, *P* = 0.8).

**Table 1:** The effect of local sex ratios on a woman’s probability of giving first birth, using a generalized linear mixed model (binary distribution, logit link function, *N* = 38,265)

Variable	Estimate	Standard error	*F* value	*P*
Intercept	−3.668	0.681		
Sex ratio	0.026	0.007	15.85	<0.0001
Age	0.105	0.004	571.04	<0.0001
Age squared	−0.006	0.001	138.51	<0.0001
Urbanity (town)	1.55	0.618	6.22	0.003
Sex ratio × urbanity (town)	−0.022	0.007	6.78	0.002
Population size	−0.001	0.022	0.02	0.94
Random effects	Variance	Standard deviation		
Year	0.024	0.007		
Municipality of residence	0.012	0.01		
Birth municipality	0.003	0.002		

Reference level for the categorical variables is in brackets.

### Age difference between spouses

Men in our sample were on average 2.4 ± 0.05 years older than their wives. We found a slight, but statistically significant result showing that the absolute age difference between spouses in our 1886 married couples (based on birth years) was positively correlated with male-biased municipality sex ratios (β = 0.0006 ± 0.003, *P* = 0.02). In other words, locations with more men were associated with higher age differences between spouses (Figure 1B; Table 2). For example, a sex ratio of 75 led to a predicted age difference of 3.25 years, whereas a sex ratio of 96 led to a predicted age difference of 3.64 years. The only other significant predictor in the model was the quadratic term of age (β = 0.005 ± 0.00008, *P* < 0.0001; Table 2). Municipality type (town/rural) did not predict age difference between spouses (β = 0.18 ± 0.87 *P* = 0.8), nor did it predict its interaction with the sex ratio (β = −0.0003 ± 0.01, *P* = 0.8). Neither the interaction between age and sex ratio nor the effect of population size (β = 0.0004 ± 0.0005, *P* = 0.5) were statistically significant (β = −0.002 ± 0.03, *P* = 0.9).

**Table 2:** Effect of local sex ratio on the age difference between spouses using a generalized linear mixed model (negative binomial distribution, log link function, *N* = 1886)

Variable	Estimate	Standard error	*F* value	*P*
Intercept	0.61	0.23		
Sex ratio	−0.006	0.003	5.54	0.02
Age	−0.0	0.051	0.81	0.4
Age squared	0.005	0.0008	42.19	<0.0001

Residuals of the model did not correlate with sex ratio, suggesting that changes in the local sex ratio were not reflected in increases or decreases in the variance of spousal age difference (β = 0.0002 ± 0.003, *P* = 0.95; Supplementary Figure 1.).

### Dispersal probability

Nearly half (47%) of the 7179 study women moved at least once during the 30,847 observation periods, and 3.5% did so at least three times. We found that sex ratio within the municipality of residence was associated with the likelihood of dispersal (β = 0.013 ± 0.003, *P* < 0.0001; Figure 1C; Table 3). An odds ratio of 1.01 indicates that a one unit increase in the sex ratio toward a less female-biased community, for example, from 88 to 89 was associated with a 1% higher probability that a woman would disperse. This is opposite to the prediction that women would be more likely to move from places with an abundance of females to areas with more males. Municipality type had a larger effect and women were 26% more likely to move away from rural areas than from towns (β = 0.2336 ± 0.063, *P* < .0001). However, women’s tendency to disperse less from female-biased municipalities was similar in both rural and urban areas, indicated by the statistically nonsignificant interaction between sex ratio and municipality type which was ultimately removed from the final model(β = 0.004 ± 0.008, *P* = 0.66). The probability to disperse decreased after the end of the war by 16.5% for each passing year (β = −0.180 ± 0.009, *P* < 0.0001). Women were also less likely to move from larger populations (β = −0.043 ± 0.021, *P* = 0.04) and were slightly more likely to disperse from municipalities where the proportion of evacuees in the population was larger (odds ratio 1.008, β = −0.008 ± 0.002, *P* < 0.0001). The number of previous peace-time movements suggest that the least likely to disperse were those women who had already moved once compared to those who had not moved at all (odds ratio 0.93), whereas those who had already moved twice or three times were more likely to move again compared to those who had not moved at all (odds ratios 1.12 and 1.99). Age was not statistically significant predictor for dispersal (β = −0.004 ± 0.004, *P* = 0.3).

**Table 3:** Effect of local sex ratio on women’s probability to disperse, using a generalized linear mixed model (binary distribution, logit link function, *N* = 30,847)

Variable	Estimate	Standard error	*F* value	*P*
Intercept	−2.2451	0.362		
Sex ratio	0.013	0.003	23.18	<0.0001
Evacuee %	0.008	0.002	23.84	<0.0001
Municipality type (town = reference)	0.234	0.063	13.88	<0.001
Years from peace	−0.180	0.009	388.04	<0.0001
Population size	−0.043	0.021	4.22	0.04
Times moved (0=reference)			18.85	<0.0001
1	−0.072	0.051		
2	0.11	0.09		
3+	0.69	0.133		
Random effects	Variance	Standard deviation		
Person ID	0.312	0.062		

Reference level for categorical variables within brackets.

## DISCUSSION

Our results demonstrate how behaviors and conditions combine to generate an “urban fertility trap,” and may provide novel perspectives to understanding the fertility dynamics of many urbanized and developing countries of today. We took advantage of a unique natural experiment where an entire population of Karelians was forcibly displaced during World War II into other local Finnish populations of varying size and sex ratio, to test how annually varying local sex ratios are linked to marriages, reproductive histories, and dispersal in rural and urban settings. We found partial support for the prediction that women would be more likely to have their first birth if the local sex ratio was less female-biased. Our results show that more female-biased sex ratio was linked to women’s probability of having their first child in towns, but not in rural areas. This difference suggests that the cultural landscape relating to family formation can differ between rural and urban areas. We cannot say with our data which are the factors driving the difference between rural and urban areas. However, we can speculate on some differences between rural and urban areas that might contribute to our findings. For example, in rural areas, small-scale farming was the most common profession and the nature of this work requires the labor of both a husband and his wife. Therefore, rural men may have been more likely to commit to a relationship even in areas where females were more abundant (also see: Guttentag and Secord 1983). Also, rural areas were not as female-biased, and had likely more traditional attitudes toward family formation and relationships (e.g., Lindberg 1955). However, both evolutionary (Schacht and Borgerhoff Mulder 2015) and market theories (Guttentag and Secord 1983) on the effects of sex ratios on pair formation were supported in female-biased urban areas where women were less likely to have their first child the more the sex ratio was biased toward females. In these areas, men may have been less willing to commit to a relationship.

Our findings showing that 1) women are less likely to give birth in female-biased environments together with 2) our failure to detect negative association between sex ratio and age differences between spouses suggest that women were largely inflexible in their choice of mates. This inflexibility may be a consequence of societal taboos for out-of-wedlock births as was the case in Finland in this time period (Lindberg 1955), and it is possible that women in female-biased environments would be more open to short-term sexual relationships or even polygamy if it was more acceptable (Josephson 2002). Although a lower age difference between partners is generally seen as an indication of a more egalitarian partnership and is frequently viewed as being more preferable for women (Kolk 2015), in female-biased populations, it is likely that less desirable men would be better able to find a mate if women were more flexible with their mating criteria and were increasingly willing to accept men who were either much older or younger than them. However, we found no evidence of this. There is also the possibility that the evacuee status of women in our study decreased their attractiveness as partners and this may be especially true in female-biased environments. Indeed, there is some evidence suggesting that evacuees were less desirable as partners by members of the host community after the war (Waris et al. 1952). Still, our data also show that evacuees were successful in reproducing in environments where more males were available which raises the question of why women did not move to improve their chances of finding a spouse through dispersal.

Because Karelian evacuees were in a position where there were fewer factors tying them to their new municipalities than locals, we predicted that female evacuees would attempt to escape unfavorable sex ratios by dispersing. However, contrary to our expectations, women were slightly more likely to move away from less female-biased populations which indicate that decisions to move were not motivated by opportunities to find a mate. One simple explanation for this result is that single women in rural areas need to find employment elsewhere because of the limited capacity of an agricultural economy to employ single women. In other words, these women may have been drawn away from rural areas by the greater employment opportunities of urban centers. For example, in post-war Finland, the textile industry created new jobs generating a strong draw for women to move into industrialized areas. Indeed, we found that women were more likely to disperse from rural areas than from towns, which is a reflection of massive urbanization in Finland after World War 2. Prioritizing working over marriage and family can also be viewed as a choice of women, which can in turn be influenced by the perceived population sex ratio and the availability of suitable mates. Indeed, one experiment showed that a female-biased sex ratio increased women’s desire for a career (Durante et al. 2012). Furthermore, it is possible that we do not see the expected effects of sex ratios on movement decisions simply because individuals’ perception of local sex ratios may not be accurate, at least in urban environments (Gilbert et al. 2016). In our analyses, the unit of population was the whole municipality, but it remains possible that the relevant social group for each individual in terms of social interaction and mate choice can be more localized.

Our results show that the likelihood of reproducing was strongly influenced by local sex ratio but that this relationship differed between rural and urban environments. While the probability of having a first child was substantially lower in urban areas where women outnumbered men, sex ratios did not seem to have any association on this likelihood in rural areas. This finding may provide much needed insight into the reasons underlying the fertility decline in many urban areas today. While several studies have examined the effect of sex ratios on mate choice (e.g., Schacht and Borgerhoff Mulder 2015), to our knowledge, there is very little individual level research that has considered the effect of skewed sex ratios on reproduction itself (Lainiala and Miettinen 2013; but see Uggla and Mace 2016, and Schacht and Smith 2017) despite the fact that it may be an important factor in studying the effects of urbanization on fertility rates, especially when migration to urban centers is female-biased.

Our findings showing a combination of a migration toward female-biased urban centers and a seeming lack of flexibility in mate choice by women result in an “urban fertility trap.” Migration of women toward cities can result from the fact that they receive increased opportunities for work, which in turn allows them to maintain independence while pursuing opportunities for education and social advancement. However, waiting for a suitable mate and even foregoing reproduction altogether may be perceived as worth the risk when weighed against the heavy burden of childbearing and raising children. Despite this possibility of missing out on reproduction altogether, we found that women did not move to centers with higher male sex ratios. Doing so would have improved their chances of finding a mate and reproducing. When viewed from the individual level, this situation forms a “trap” whereby women are initially attracted to urban centers for work, education and independence, but are then faced with a highly competitive mating market which significantly reduces their prospects of reproducing. Together, these individual decisions and conditions can then result an overall reduction in fertility in these female-biased urban environments.

Further nuances regarding the effect of sex ratios on the fertility decisions of women can also arise. Previous research, for example, suggests that socioeconomic status may also play a role in how women adjust their reproductive strategy when faced with female-biased sex ratios. Chipman and Morrison (2013) analyzed the reproductive behavior of urban residents in 2005–2007 in England and found that when faced with female-biased sex ratios, women from poor areas gave birth earlier whereas wealthier women gave birth later. Uggla and Mace (2016) also found that the onset of reproduction was earlier for women in female-biased environments in contemporary Northern Ireland and suggested a proximate explanation for this where women are more open to the prospect of unprotected sex when competition for men is high. Our sample is exceptional in that the evacuees were recent arrivals in both the urban and rural areas and due to the loss of most of their possessions had relatively little differences in wealth among them. The different social norms with respect to giving birth outside of marriage in post-war Finland versus those of modern day Western societies undoubtedly also affect reproductive decisions. A shortcoming of our study is that our measure of sex ratio includes all age classes and marital statuses and if the age structure in towns and rural areas differs considerable, our measure of sex ratio might not reflect the availability of marriage partners in a similar way in each location; in other words, if age structure was younger in urban areas, it would reflect the sex ratio of potential partners better than the sex ratio of rural areas. We therefore welcome more nuanced future studies comparing the urban and rural sex ratio effects on female behavior in different contexts.

## CONCLUSIONS

In this study, we have shown how mate choice, sex ratios, and migration are all interlinked and have important demographic and societal implications. Our study has shown that in a post-war, female-biased environment, young women who had not yet given birth migrated toward urban centers and away from areas (urban or rural) where men were more plentiful, presumably to areas where work was more available to women. In these urban areas where female-biased sex ratios were at their highest, women were less likely to start reproduction. Our results, however, demonstrated that there was no increase in the variance in partner age in areas where female sex ratios were highest, suggesting that women were not very flexible in their choice of mates. We propose that this combination of behaviors and events can have far reaching consequences on overall fertility in urban centers. Although the population studied here is a historic one, our findings can be applicable to present day urban environments. For example, in Finland, the decline has been quite dramatic—total fertility rate (TFR) has declined from 1.87 to 1.41—that is, by 25% between 2010 and 2018 (Official Statistics of Finland 2018) while at the same time migration toward the capital region has continued and the sex ratio is increasingly skewed toward females (Wiest et al. 2013). Lack of a suitable partner is one of the main reasons for childlessness among 25–34 year old men and women in Finland (Miettinen and Rotkirch 2012) and an imbalance in sex ratios among different socioeconomic classes might be one reason for this. This demographic situation is not unique to Finland, and the urban fertility trap scenario presented here may be common to many modern urban centers worldwide. Therefore, these results have implications for understanding fertility trends globally.

## Supplementary Material

arab007_suppl_Supplementary_Material

## Data Availability

Analyses reported in this article can be reproduced using the data provided by Pettay et al. (2021).

## Notes

We thank Kone Foundation (J.L., J.E.P., and R.L.), the Academy of Finland (grant 292368; J.E.P. and V.L.), NEFER (decision 321264), and the INVEST research flagship 320162 (R.L.) for funding. We are also grateful to Simon Chapman and Salman Kasi for help with the data. We thank four anonymous reviewers for their constructive remarks. The authors declare no competing interests.
