# Correction to: Female-biased sex ratios in urban centers create a “fertility trap” in  post-war Finland

**DOI:** 10.1093/beheco/arac030

**Published:** 2022-04-26

**Authors:** Jenni E Pettay, Virpi Lummaa, Robert Lynch, John Loehr

**Affiliations:** Department of Social Research, University of Turku, 20014 Turku, Finland; Department of Biology, University of Turku, 20014 Turku, Finland; Pennsylvania State University, State College, PA, USA; Faculty of Biological and Environmental Sciences, Lammi Biological Station, University of Helsinki, 16900 Lammi, Finland

In the originally published version of this manuscript, the values in Table 2 were incorrect. The figures quoted in the main text were correct. Table 2 has been corrected online.

