# Supplementary material for: Female-biased sex ratios in urban centers create a “fertility trap” in post-war Finland
Source: Behav Ecol. 2021 Mar 18;32(4):590–8. doi: 10.1093/beheco/arab007 (PMC8442939; doi:10.1093/beheco/arab007)
Supplement: arab007_suppl_Supplementary_Material [file arab007_suppl_supplementary_material.docx]

Supplementary material for:

Female-biased sex ratios in urban centres create a ‘fertility trap’ in post war Finland

Supplementary figure 1. Residuals from the model analysing the relationship between spousal age differences shows equal variances regardless of sex ratio.
